# Is Single Nucleotide Polymorphism *ADIPOQ* (NM_004797.4):c.214+62G>T (rs1501299) Associated With Uterine Leiomyomas? A Pilot Study

**DOI:** 10.3389/pore.2021.1609966

**Published:** 2022-02-18

**Authors:** Jan Bieńkiewicz, Beata Smolarz, Miłosz Wilczyński, Anna Stepowicz, Grzegorz Jabłoński, Anna Obłękowska, Andrzej Malinowski, Hanna Romanowicz

**Affiliations:** ^1^ Department of Operative Gynecology, Endoscopy and Gynecologic Oncology, Polish Mother’s Memorial Hospital-Research Institute, Lodz, Poland; ^2^ Laboratory of Cancer Genetics, Department of Clinical Pathology, Polish Mother’s Memorial Hospital-Research Institute, Lodz, Poland; ^3^ Department of Obstetrics, Perinatology and Gynecology, Polish Mother’s Memorial Hospital-Research Institute, Lodz, Poland; ^4^ Department of Operative and Endoscopic Gynecology, Medical University of Lodz, Lodz, Poland; ^5^ Department of Clinical Pathology, Polish Mother’s Memorial Hospital-Research Institute, Lodz, Poland

**Keywords:** single nucleotide polymorphism, obesity, uterine leiomyomas, uterine fibroids, c.276 G>T, adiponectin, ADIPOQ, (NM_004797.4):c.214+62G>T

## Abstract

**Objective:** Although polymorphisms of adiponectin gene (*ADIPOQ*) in obesity-related conditions have been the target of research efforts, little is known about this genetic marker in uterine leiomyomas. The aim of this pilot study was to analyze the frequencies of alleles and genotypes of Single Nucleotide Polymorphism *ADIPOQ* (NM_004797.4):c.214+62G>T (rs1501299) and to correlate it with the risk of uterine fibroids.

**Study Design:** The Test Group comprised 90 women treated surgically for uterine leiomyomas in the Department of Operative Gynecology, Endoscopy and Gynecologic Oncology, Polish Mother’s Memorial Hospital-Research Institute. 90 disease-free individuals were used as Controls. Patients within both groups were additionally stratified into lean, overweight and obese, according to Body Mass Index. Statistical analysis was performed between the two major groups and, furthermore, within the abovementioned subgroups.

**Results:** The study revealed no statistically significant differences in the distribution of alleles and genotypes of SNP *ADIPOQ* (NM_004797.4):c.214+62G>T (rs1501299) between the two main groups. A weak correlation within distributions of alleles was observed between obese Test Patients and lean Controls.

**Conclusion:** This pilot study has revealed no association between SNP *ADIPOQ* (NM_004797.4):c.214+62G>T (rs1501299) and uterine fibroids. Further studies on larger groups are warranted to elucidate whether this SNP may be correlated with uterine leiomyomas.

## Introduction

Uterine fibroids (UFs), often referred to as uterine leiomyomas, are highly prevalent benign smooth muscle tumors of the uterus. Lifetime risk of this condition varies according to sources, but may reach as high as 75% [1, 2]. Numerous theories have been proposed on how and why UFs develop, however, their pathogenesis has not been yet clarified satisfactorily and is not fully understood [1–3]. Several studies have shown that the prevalence of UF is highest in females aged 26–30 and directly corresponds to overall body adiposity expressed by Body Mass Index (BMI) [4, 5]. The latter correlation is consistent with estrogen-dependency doctrine, which has gained particular attention, where excess fatty tissue eventually leads to elevated estrogen levels by facilitating peripheral conversion of androgen to estrogen.

Regrettably, not much scientific attention has been focused on understanding genetics and epigenetics of UFs. Välimäki et al. [6] however, have published a large study on more than 15,000 uterine fibroids cases and almost 400,000 controls. The authors have pointed 22 loci which displayed a genome-wide significant susceptibility to leiomyomagenesis. These genes were linked with two distinct biological processes: genome stability and genitourinary development. In other studies, it has been suggested, that the vast majority of UFs can be subject to one of four major categories of mutations: MED 12 mutations, FH inactivation, COL4A6-COL4A5 deletions, or HMGA2 overexpression [7–9]. However, still little is known about genetic polymorphism in UFs.

On the contrary, widespread research efforts have been put into exploring obesity and genetic polymorphism in obesity-related genes. This trend could be explained by the modern approach to adipose tissue, which is not only the energy storage compartment but rather a vital endocrine organ [10, 11]. Among adipokines, two major players have gained particular attention: adiponectin and leptin with approximately 8,500 and 16,000 titles in PubMed database, respectively (as of October 2021). Adiponectin, which is synthesized only in adipocytes, is inversely correlated with overall body fat content [12, 14] and exerts a protective influence on such conditions as: diabetes, insulin resistance or even endometrial cancer [13–19].

The issue of genetic polymorphism in adiponectin gene and its association with obesity has been widely studied. Synonymous Single Nucleotide Polymorphism *ADIPOQ* (NM_004797.4):c.214+62G>T (rs1501299) has been thoroughly analyzed in up-to-date literature on the abovementioned matter and its correlation with BMI is generally hypothesized [20–25]. The findings are however incoherent probably due to various factors including ethnicity and geographical distribution of tested subjects [26–28]. Moreover, the frequency of genetic variants also differs across populations: Minor Allele Frequency (MAF) reaches from 0, 15 to 0, 34 [29]. Although the exact biological significance of this SNP and the specific mechanisms how its variants alter the levels and activity of adiponectin remain unclear, it is postulated that this marker may potentially affect transcriptional activity or splicing efficiency [30].

In our earlier study [32] in which we analyzed the role of *ADIPOQ* (NM_004797.4):c.214+62G>T (rs1501299) in Endometrial Cancer, patients treated for uterine leiomyomas were used as Controls. The selection of the control group, and thus the whole study design, was then challenged by some reviewers, as regrettably, the correlation between this SNP and uterine fibroids had not been established at that point. Encouraged by that and to elucidate whether this DNA marker has any influence on uterine leiomyomas, we have decided to go a step further and examine disease-free controls. Until now, to our best knowledge *ADIPOQ* (NM_004797.4):c.214+62G>T (rs1501299) still has not been analyzed in uterine leiomyomas. The aim of this study was to analyze the frequencies of alleles and genotypes of Single Nucleotide Polymorphism *ADIPOQ* (NM_004797.4):c.214+62G>T (rs1501299) and to correlate it with the risk of uterine fibroids.

## Study Design

### Patients

The Test Group comprised 90 women treated surgically for UFs in the Department of Operative Gynecology, Endoscopy and Gynecologic Oncology, Polish Mother’s Memorial Hospital-Research Institute, Lodz, Poland. 90 healthy age- and BMI-matched individuals were used as Controls. DNA of the latter was provided by BioBank Laboratory (University of Lodz, Poland). For further statistical investigation of potential role of SNP *ADIPOQ* (NM_004797.4):c.214+62G>T (rs1501299) in obesity and UFs, both groups have been stratified accordingly to total body fat content (using BMI—Body Mass Index—as a marker), into lean (BMI < 25), overweight (25 ≤ BMI < 30) and obese (BMI ≥ 30) and thus six groups (30 patients each) were obtained.

Test Group 1 (TG1)—BMI < 25 (n = 30)
Test Group 2 (TG2)—25 ≤ BMI < 30 (n = 30)
Test Group 3 (TG3)—BMI ≥ 30 (n = 30)
Controls 1 (C1)—BMI < 25 (n = 30)
Controls 2 (C2)—BMI 25 ≤ BMI < 30 (n = 30)
Controls 3 (C3)—BMI ≥ 30 (n = 30)

The summaries of both Test Group and Controls are presented in Tables 1, 2.

**TABLE 1:** Test group.

	Age: mean (median, SD)	BMI: mean (median, SD)
TG1	58.6 (54; ± 12.5)	22.7 kg/m^2^ (23.3 kg/m^2^; ± 1.6)
TG2	60.9 (57.5; ± 11.2)	27.9 kg/m^2^ (28.1 kg/m^2^; ± 1.3 )
TG3	63.8 (64; ± 9.5)	34.9 kg/m^2^ (34.5 kg/m^2^; ± 1.9)
In total	61.1 (62; ± 11.2)	28.6 kg/m^2^ (28.1 kg/m^2^; ± 5.2)

**TABLE 2:** Controls.

	Age: mean (median; SD)	BMI: mean (median; SD)
C1	54.3 (54; ± 4.2)	22.6 kg/m^2^ (23.1 kg/m^2^; ± 1.9)
C2	57.5 (56; ± 4.6)	27.2 kg/m^2^ (27.5 kg/m^2^; ± 0.2)
C3	57.5 (55; ± 6.2)	33.4 kg/m^2^ (33.2 kg/m^2^; ± 2.5)
In total	56.4 (55; ± 5.3)	27.9 kg/m^2^ (27.5 kg/m^2^; ± 5.0)

Due to a well-established role of the investigated SNP in metabolic disorders and its potential significance in cancer development, a history of any such comorbidity was an exclusion criterion of the study. The study received internal funding grant from Polish Mother’s Memorial Hospital-Research Institute, Lodz, Poland (grant no. 2015/VII/29-MN).

### Genotype Determination

To investigate the Test Group, DNA was retrieved from archival postoperative specimens (paraffin blocks stored in the Department of Clinical Pathology, Polish Mother’s Memorial Hospital-Research Institute, Lodz, Poland). Tissue samples, after original fixation in formaldehyde and embedding in paraffin, were microtome-sectioned at thicknesses of 5 µm and stained with hematoxylin and eosin. Then, the slices were placed in Eppendorf^®^ micro test tubes, shaken five times with xylene which was followed by 3-minute-long centrifugation (14,000 RPM) after each shaking. The sediment was lavaged in 96% ethanol, again centrifuged for 3 min and then dried in 37°C. DNA was extracted from the material by DNeasy Blood & Tissue Kit (Qiagen, Germany) according to manufacturer’s manual. DNA samples of Controls were provided by BioBank Laboratory (University of Lodz, Poland). For both Test Group and Controls, Polymerase Chain Reaction - Restriction Fragment Length Polymorphism (PCR-RFLP) was used to determine the genotypes of SNP *ADIPOQ* (NM_004797.4):c.214+62G>T (rs1501299). Following primers were applied (Polgen, Poland):

Forward: 5′TCT​CTC​CAT​GGC​TGA​CAG​TG3′
Reverse: 5′AGA​TGC​AGC​AAA​GCC​AAA​GT3′

The PCR-RFLP was completed in PTC-100 TM unit (MJ Research, INC, Waltham, MA, United States). The amplification was performed in 50 μl of reaction mixture which consisted of: genomic DNA, PCR buffer (TaKaRa, Japan), dNTP (TaKaRa, Japan), Taq Polymerase (TaKaRa, Japan), primers (Polgen, Poland) and deionized H_2_O. PCR cycler conditions were as follows: 95°C for 30 s, 62°C for 30 s and 72°C for 30 s—repeated for 35 cycles. The product (set in 20 μl of reaction mixture) was incubated in 65°C for 14 h with restriction enzyme (*Bsm*I, New England BioLabs Inc., United States). PCR-RFLP products were then electrophoresed in a 2% agarose gel (Sigma, Saint Louis, United States) and visualized by ethidium bromide staining (Sigma, Saint Louis, United States). DNA Ladder 100 bp (Polgen, Poland) was used as mass ruler. Agarose gel was studied in ultraviolet light (Kodak Edas 290). The reaction produced fragments of 468bp (homozygous: GG), 468, 320 and 148 bp (heterozygous: GT) and 320 and 148 bp (homozygous: TT).

### Statistical Analysis

χ2-test was used to assess the departure from Hardy-Weinberg equilibrium. Genotype and allele frequencies in Test Group and Controls were compared by χ2-test. Specific risks were expressed as odds ratios (ORs) with associated 95% confidence intervals (CIs) and adjusted to the logistic regression model. *p*-Values < 0.05 were considered significant.

## Results

**FIGURE 1:**
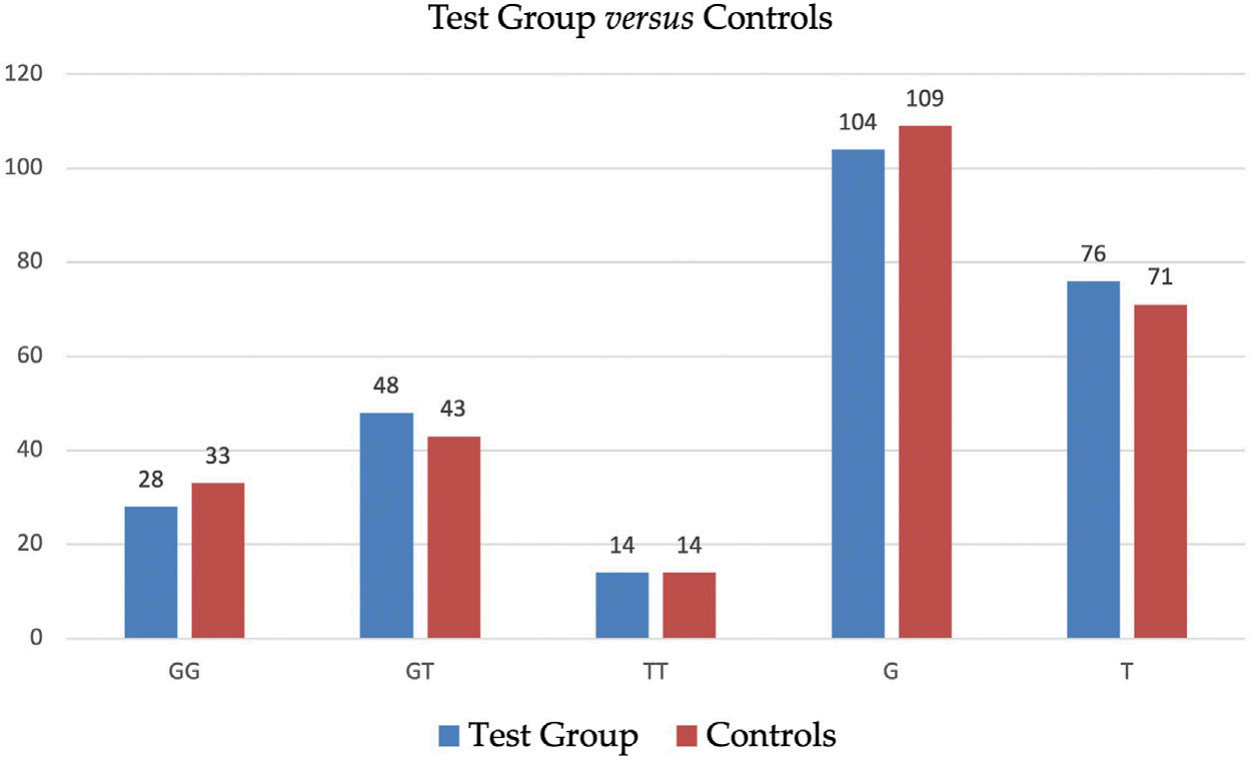
Distribution of genotypes and alleles of SNP ADIPOQ (NM_004797.4):c.214+62G>T (rs1501299) in Test Group and in Controls.

Table 3 displays the detailed distribution of genotypes and alleles of SNP *ADIPOQ* (NM_004797.4):c.214+62G>T (rs1501299) in Test Group and in Controls with a further subdivision accordingly to BMI. The statistical analysis did not reveal significant differences in the distribution of genotypes and alleles of SNP *ADIPOQ* (NM_004797.4):c.214+62G>T (rs1501299) between Test Group and Controls—see Table 4. Graphic illustration is provided in Figure 1.

**TABLE 3:** Detailed distribution of genotypes and alleles of SNP *ADIPOQ* (NM_004797.4):c.214+62G>T (rs1501299) in Test Group and in Controls.

	Test group (n = 90)						Controls (n = 90)					
	TG1 (n = 30)		TG2 (n = 30)		TG3 (n = 30)		C1 (n = 30)		C2 (n = 30)		C3 (n = 30)	
	n	%	n	%	n	%	n	%	n	%	n	%
Genotype												
GG	5	17	12	40	11	37	11	37	12	40	10	33
GT	19	63	14	47	15	50	16	53	11	37	16	53
TT	6	20	4	13	4	13	3	10	7	23	4	13
Total	30	100	30	100	30	100	30	100	30	100	30	100
Allele												
G	29	48	38	63	37	62	38	63	35	58	36	60
T	31	52	22	37	23	38	22	37	25	42	24	40
Total	60	100	60	100	60	100	60	100	60	100	60	100

**TABLE 4:** Distribution of genotypes and alleles of SNP *ADIPOQ* (NM_004797.4):c.214+62G>T (rs1501299) in Test Group and in Controls.

Genotype/Allele	Test group (n = 90)		Controls (n = 90)		OR (95% PU)^a^	*p* ^b^
	n	%	n	%		
GG	28	31	33	37	**1.00 Ref.** **^c^**	
GT	48	53	43	48	0.76 [0.39–1.45]	0.507
TT	14	16	14	16	0.84 [0.34–2.07]	0.887
G	104	58	109	61	**1.00 Ref.** **^c^**	
T	76	42	71	39	0.89 [0.59–1.35]	0.671

a Odds ratio analysis [OR, odds ratio; CI, confidence interval 95%].

b For the dparture from Hardy-Weinberg equilibrium.

c Reference wild allele.

**FIGURE 2:**
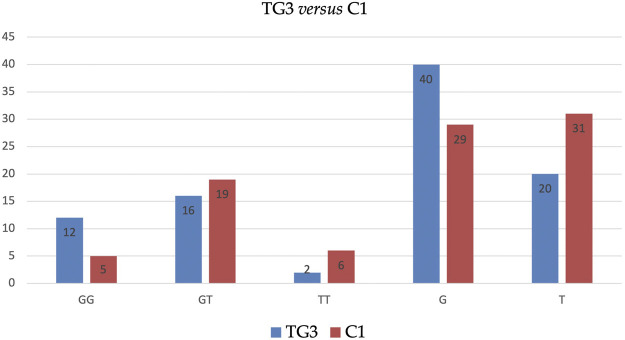
Distribution of genotypes and alleles of SNP *ADIPOQ* (NM_004797.4):c.214+62G>T (rs1501299) in TG3 *vs.* C1.

Similarly, BMI-adjusted analysis of subgroups (i.e., TG1 *vs*. C1, TG2 *vs*. C2, TG3 *vs*. C3) has not revealed any statistically significant outcomes. However, the analysis revealed that allele G in TG3 is significantly more frequent (67 *vs*. 48%), and allele T in these patients is significantly less frequent (33 *vs*. 52%) than in C1 (see Table 5 and Figure 2).

**TABLE 5:** Distribution of genotypes and alleles of SNP *ADIPOQ* (NM_004797.4):c.214+62G>T (rs1501299) in TG3 *vs.* C1.

Genotype/Allele	TG3 (n = 30)		C1 (n = 30)		OR (95% CI)^a^	*p* ^b^
	n	%	n	%		
GG	12	40	5	17	**1.00 Ref.^c^**	
GT	16	53	19	63	0.35 [0.10–1.21]	0.163
TT	2	7	6	20	**0.14 [0.02–0.94]**	**0.043**
G	40	**67**	29	48	**1.00 Ref.^c^**	
T	20	**33**	31	52	**0.47 [0.22–0.97]**	**0.042**

a Odds ratio analysis [OR, odds ratio; CI, Confidence Interval 95%].

b χ2 for the departure from Hardy-Weinberg equilibrium.

c Reference: wild allele.

In further analysis, correlations within the main groups were investigated: lean women with UFs (TG1) were tested *versus* obese ones (TG3) and lean controls (C1) *versus* obese controls (C3). Alike, no statistically significant findings were observed within the two abovementioned two comparisons (see Tables 6, 7).

**TABLE 6:** Distribution of genotypes and alleles of SNP *ADIPOQ* (NM_004797.4):c.214+62G>T (rs1501299) within Test Group: lean (TG1, BMI < 25) *vs.* obese (TG3, BMI ≥ 30).

Genotype/Allele	Lean (TG1) (n = 30)		Obese (TG3) (n = 30)		OR (95% CI)^a^	*p* ^b^
	n	%	n	%		
GG	5	17	11	37	**1.00 Ref.^c^**	
GT	19	63	15	50	2.78 [0.79–9.78]	0.186
TT	6	20	4	13	3.30 [0.63–17.16]	0.150
G	29	48	37	62	**1.00 Ref.^c^**	
T	31	52	23	38	1.71 [0.83–3.55]	0.198

a Odds ratio analysis [OR, odds ratio; CI, Confidence Interval 95%].

b χ2 for the departure from Hardy-Weinberg equilibrium.

c Reference: wild allele.

**TABLE 7:** Distribution of genotypes and alleles of SNP *ADIPOQ* (NM_004797.4):c.214+62G>T (rs1501299) within Controls: lean (C1, BMI < 25) *vs.* obese (C3, BMI ≥ 30).

Genotype/Allele	Lean (C1) (n = 30)		Obese (C3) (n = 30)		OR (95% CI)^a^	*p* ^b^
	n	%	n	%		
GG	11	37	10	33	**1.00 Ref.^c^**	
GT	16	53	16	53	0.91 [0.30–2.73]	0.920
TT	3	10	4	13	0.68 [0.121–3.82]	0.499
G	38	63	36	60	**1.00 Ref.^c^**	
T	22	37	24	40	0.86 [0.41–1.81]	0.841

a Odds ratio analysis [OR, odds ratio; CI, Confidence Interval 95%].

b χ2 for the departure from Hardy-Weinberg equilibrium.

c Reference: wild allele.

## Discussion

As mentioned in the Introduction, this research was partly motivated by our previous study [31] and some concerns raised then over the selection of controls. By that time, and until this day, the role of SNP *ADIPOQ* (NM_004797.4):c.214+62G>T (rs1501299) in UFs has not been elucidated. To explore this issue, in the current study we enrolled some new samples from disease-free age- and BMI-matched individuals and conducted a Pilot Study on this issue. This study extension was enabled by the newly established cooperation between our Institution and BioBank (University of Lodz, Poland), which provided us with suitable disease-free samples. This Pilot Study revealed no statistically significant differences in the distribution of genotypes and alleles of Single Nucleotide Polymorphism *ADIPOQ* (NM_004797.4):c.214+62G>T (rs1501299) between the main groups, which allows to draw a preliminary conclusion, that uterine leiomyomas are not correlated with this SNP (see Table 4). However, a statistically significant correlation was observed when obese Test Group patients were juxtaposed with lean Controls (TG3 *vs.* C1), where allele G proved to be more frequent, while allele T less frequent in the former group (Table 5 and Figure 2). It is noteworthy, that this result concerns alleles alone with no impact on the distribution of complete genotypes. Furthermore, this association is rather weak, and no definite conclusions should be drawn basing on this finding only. Besides, this weak association can be easily challenged, as it was solely observed between groups that differ not only by uterine fibroids’ presence, but—more importantly—by total body fat content (expressed by BMI) which may play a role in the distribution of genotypes and alleles of the studied SNP [21–26]. Interestingly, in our research the distribution of genotypes and alleles of the studied SNP was not associated with BMI (Tables 6, 7) which rather suggests that the observed weak correlation should not be attributed to overall body adiposity.

Moreover, one must mention the limitations of our research. To begin with, the sample number is low and may be considered quantitively unsatisfactory to make final conclusions in a genetic study [32, 33]. Furthermore, an issue of studying a single SNP may also be raised as an obvious drawback here. Therefore, taking into consideration the preliminary data of our pilot study, we believe that further research in this field is needed.

## Conclusion

According to our Pilot Study, SNP *ADIPOQ* (NM_004797.4):c.214+62G>T (rs1501299) is not associated with uterine leiomyomas. Taking into consideration some clear limitations of our research, conclusions should be drawn with caution. Further research on larger groups is warranted to improve the credibility of our finding.

## Data Availability

The raw data supporting the conclusions of this article is stored at the Institution and will be made available by the authors upon request.
